# Changes in Iris Perfusion Following Scleral Buckle Surgery for Rhegmatogenous Retinal Detachment: An Anterior Segment Optical Coherence Tomography Angiography (AS-OCTA) Study

**DOI:** 10.3390/jcm9041231

**Published:** 2020-04-24

**Authors:** Rossella D’Aloisio, Pasquale Viggiano, Enrico Borrelli, Mariacristina Parravano, Aharrh-Gnama Agbèanda, Federica Evangelista, Giada Ferro, Lisa Toto, Rodolfo Mastropasqua

**Affiliations:** 1 Ophthalmology Clinic, Department of Medicine and Science of Ageing, University G. D’Annunzio Chieti-Pescara, 66100 Chieti, Italy; ross.daloisio@gmail.com (R.D.); borrelli.enrico@yahoo.com (E.B.); gnamaomer@tiscali.it (A.-G.A.); federica.evan@hotmail.com (F.E.); giadaferro90@gmail.com (G.F.); l.toto@unich.it (L.T.); 2 IRCCS Fondazione G.B.Bietti per lo Studio e la Ricerca in Oftalmologia ONLUS, 00198 Roma, Italy; mcparravano@gmail.com; 3 Facoltà di Medicina e Chirurgia dell’Università di Modena e Reggio Emilia, 41121 Modena, Italy; rodolfo.mastropasqua@gmail.com

**Keywords:** optical coherence tomography angiography, rhegmatogenous retinal detachment, scleral buckling, anterior-segment

## Abstract

Purpose: To investigate iris vasculature changes following scleral buckling (SB) surgery in eyes with rhegmatogenous retinal detachment (RRD) with anterior-segment (AS) optical coherence tomography angiography (OCTA). Methods: In this prospective study, enrolled subjects were imaged with an SS-OCTA system (PLEX Elite 9000, Carl Zeiss Meditec Inc., Dublin, CA, USA). Image acquisition of the iris was obtained using an AS lens and a manual focusing adjustment in the iris using the retina imaging software. The quantitative analysis was performed in eight different iris regions: (i) superior, (ii) supero-temporal, (iii) supero-nasal, (iv) nasal, (v) temporal, (vi) inferior, (vii) infero-temporal, (viii) infero nasal which, were defined as squares with area of 1.5 mm^2^. Results: Fifteen eyes of 15 patients (six females; nine males) were included. Anterior segment optical coherence tomography angiography (AS-OCTA) parameters of the iris were statistically compared at baseline (preoperatively), 1 week, 1 month and 6 months after SB. At post-operative 1 week, perfusion density (PD) showed a significant decrease from 66.8 ± 13.2% to 58.55 ± 12.0% in the iris supero-nasal region (*p* = 0.016). However, at the 1-month follow-up visit, iris PD was significantly lower in all the analyzed iris regions, apart from the superior one. Conclusions: This study is the first description of AS-OCTA in patients undergoing SB. Our results showed a uniform reduction of the iris vessel network at 1 month after surgery, supporting the clinical use of AS-OCTA to identify early iris perfusion changes as potential predictive biomarkers of vascular disorders.

## 1. Introduction

Rhegmatogenous retinal detachment (RRD) represents a common cause of visual impairment secondary to separation of neurosensory retina from the underlying retinal pigment epithelium (RPE) [1]. The RRD development typically involves the association between posterior vitreous detachment and one or more retinal breaks causing the fluid passage through the retinal breaks into subretinal space [2,3].

Scleral buckling (SB) is still the first line surgical approach in primary RRD and for selected cases, despite the worldwide spread of vitrectomy in the last decades [4,5,6]. This procedure involves an extraocular approach aimed at restoring contact of the RPE with the detached neuroretina [1,6]. Scleral buckling is a safe technique with a low incidence of perioperative and postoperative complications, however, several studies have highlighted the occurrence of a wide range of possible complications following surgery. These include glaucoma, choroidal detachment, eye movement disorder, infection and extrusion of the buckling material, and refractive changes [2,4,6]. Furthermore, previous histopathological studies have suggested that surgical manoeuvres during scleral buckling surgery (e.g., placement of encircling bands, compression of the eyeball, and cryotherapy to the scleral vessels) may result in an interruption of the blood supply to the anterior segment of the eye [2,4,5,6,7,8,9].

The introduction of optical coherence tomography angiography (OCTA) allowed the evaluation of the retinal and choroidal vasculature in healthy and pathologic eyes [10,11,12,13,14].

,New generation OCTA devices such as anterior segment OCTA (AS-OCTA) have opened a new scenario for the diagnosis and prognosis of different ocular conditions regarding the anterior segment of the eye, for the chance to visualize and analyze AS structure and vasculature [15,16,17,18].

The aim of this study was thus to assess the iris vasculature in eyes with RRD and undergoing SB using AS-OCTA, in order to evaluate potential changes of this surgical technique on the iris perfusion. This might reveal attractive metrics that could potentially be used as a tool to identify patients at higher risk of developing anterior segment ischemia (ASI) after surgery.

## 2. Methods

### 2.1. Study Participants

In this prospective, longitudinal study, we enlisted patients with RRD scheduled for episcleral surgery at the Ophthalmology Clinic of University G. d’Annunzio, Chieti-Pescara, Italy. The Institutional Review Board (IRB) approved this study which adhered to the ethics tenets of the Declaration of Helsinki. Data acquisition and analysis was performed in compliance with protocols approved by the Ethical Committee of the Martin Luther University Halle-Wittenberg (ethical approval number 2015-18).

All subjects enlisted were imaged with the AS-OCTA PLEX Elite 9000 device (Carl Zeiss Meditec Inc.) between February 2019 and May 2019.

Fifteen patients (15 eyes) affected by both macula-involving or -not involving RRD were included. Exclusion criteria were: wide retinal breaks, retinal detachment following macular hole, breaks situated at posterior pole, proliferative vitreoretinopathy, angle closure, glaucoma diagnosis, preceding ocular surgery, prior ocular trauma, other ocular diseases, a refractive error ≤5 D (spherical equivalent).

At RRD diagnosis (baseline), all patients underwent an ophthalmologic evaluation, including the assessment of Snellen best-corrected visual acuity (BCVA), intraocular pressure (IOP), and dilated ophthalmoscopy, fundus scleroindentation with indirect ophthalmoscope. Subsequently, patients underwent AS-OCTA (PLEX Elite 9000 device; Carl Zeiss Meditec Inc.).

AS-OCTA acquisition was performed preoperatively, 1 week, 1 month and 6 months after surgical procedure. A group of age-matched healthy eyes were considered as control (*n* = 15).

Written informed consent was obtained from all participants prior to study.

### 2.2. Surgical Procedure

General anesthesia for all patients; episcleral surgery was executed in 24 hr in retinal detachment involving macular zone and in 7 days in retinal detachment not involving macular zone. The surgery involved the application of a silicon encirclement, drainage of sub-retinal fluid, intravitreal injection of tamponade with air, sulfur hexafluoride, or perfluoropropane, cryotherapy and radial sponges to close of retinal breaks in all patients.

The operations were carried out by a single surgeon (A.-G.A.), and episcleral surgery was successful when the retina appeared reattached at the end of the procedure.

### 2.3. Imaging

Subjects underwent AS-OCTA imaging using the PLEX Elite 9000 device (Carl Zeiss Meditec Inc.) which uses a swept laser source with a central wavelength of 1050 nm (1000–1100 nm full bandwidth) and operates at 100,000 A-scans per second. Image acquisition of the iris was obtained using an AS lens (+ 20 Diopters) and a manual focusing adjustment in the iris using the retina imaging software. To obtain good quality images, the examiner had to focus on the center of the region of interest, and the patient was required to avoid eye movement [17].

A full frontal 9 × 9 mm iris scan was performed. Internal artifact software was used to reduce motion artifacts. AS-OCTA scans were captured in both eyes and always by the same operator (P.V.) in a masked fashion to the patient and clinical course. Furthermore, we excluded and repeated poor quality images with a signal strength index lower than 6 (a measurement in a scale 0–10 indicating the level of retinal tissue signal with respect to the noise or background level in optical coherence tomography (OCT) data), as recommended by manufacturers.

### 2.4. Image Processing

The principal outcome was to measure the iris perfusion change in eyes with retinal detachment before and after SB.

**Figure 1:**
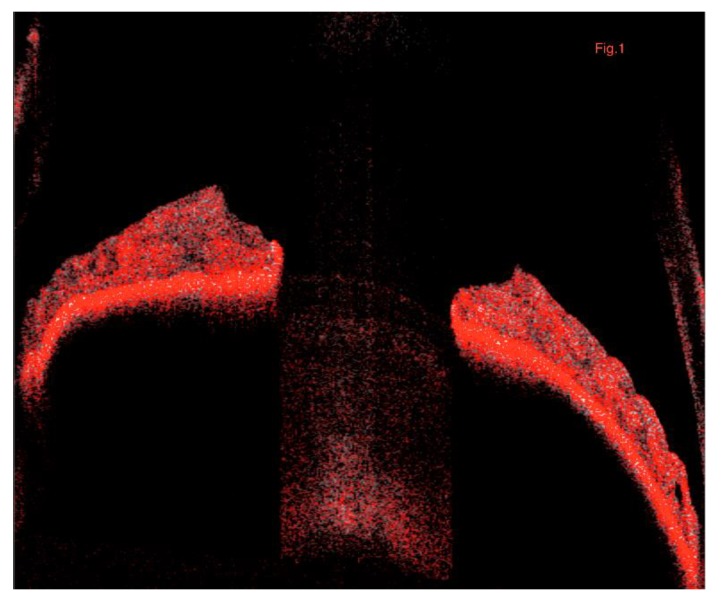
Representation of the B-scan iris image of the OCTA. The segmented boundaries include the whole iris lumen providing a representative iris vascular network image. OCTA, optical coherence tomography angiography.

All selected images were carefully visualized by two retinal specialists (L.T. and R.M.) in consensus to ascertain the correctness of segmentation and in case of erroneous recognition by the software of the position of the iris boundaries manual correction was performed using the segmentation and propagation editing tool from the device. For this reason, we manually increased the distance between the segmented boundaries in order to include the whole iris lumen and obtain a representative iris vascular network image (Figure 1).

**Figure 2:**
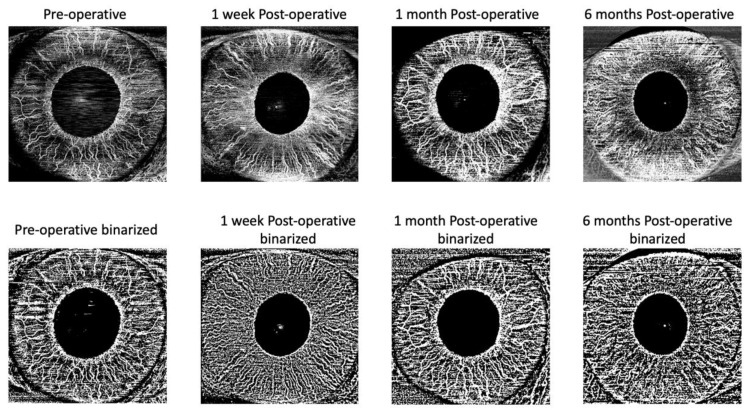
Representation of the algorithm used to process the images. The iris en face image was imported into ImageJ and the “Moments” threshold was used to binarize the iris images. Obtained images were processed with the ‘Analyze Particles’ command, in order to measure the perfusion density (PD). PD was thus calculated as a unitless proportion of the number of pixels over the threshold divided by the total number of pixels in the analyzed area.

Subsequently, for each eye, we exported the iris vascular images using PLEX Elite 9000 device (Carl Zeiss Meditec Inc.). These images were imported in ImageJ software version 1.50 (National Institutes of Health, Bethesda, MD; available at http://rsb.info.nih.gov/ij/index.html) and, the “Moments” threshold binarized the iris images (Figure 2). Subsequently, the images were processed with the, ‘Analyze Particles’ command, in order to measure the perfusion density (PD). PD was thus calculated as a unitless proportion of the number of pixels over the threshold divided by the total number of pixels in the analyzed area.

**Figure 3:**
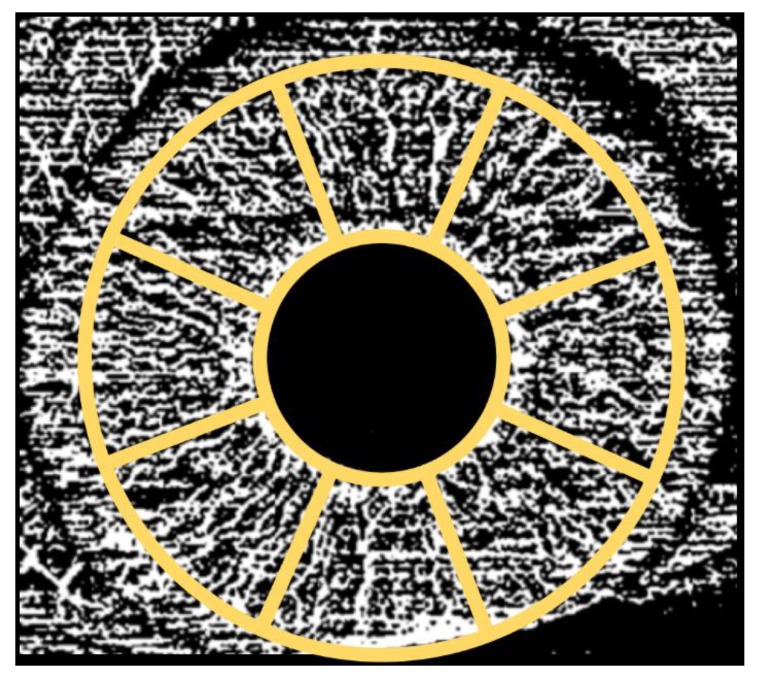
Representation of the regions used to investigate optical coherence tomography angiography variables. The quantitative analysis was performed in eight different iris regions: (i) superior, (ii) supero-temporal, (iii) supero-nasal, (iv) nasal, (v) temporal, (vi) inferior, (vii) infero-temporal, (viii) infero nasal which, were defined with a special scheme created by us.

The quantitative analysis was performed in eight different iris regions: (i) superior, (ii) supero-temporal, (iii) supero-nasal, (iv) nasal, (v) temporal, (vi) inferior, (vii) infero-temporal, (viii) infero nasal which were defined with a special scheme created by us. Every single region consists in a 450-mm^2^ area. (Figure 3).

### 2.5. Statistical Analysis

All quantitative variables were reported as mean and standard deviation (SD) in the Results section and in the tables. To detect departures from normality distribution, Shapir o–Wilk’s test was performed for all variables. Wilcoxon signed-rank test with correction for multiple comparison was applied to compare baseline values with each follow-up controls. Statistical calculations were performed using Statistical Package for Social Sciences (version 20.0, SPSS Inc., Chicago, IL, USA). The chosen level of statistical significance was *p* < 0.05.

## 3. Results

### 3.1. Characteristics of Subjects Included in the Analysis

Fifteen eyes of 15 patients (six females; nine males) were included. RRD affected 9 (60%) right and 6 (40%) left eyes. Mean ± SD age was 50.3 ± 17.6 years. Mean ± SD axial length was 25.2 ± 1.5 mm (Table 1). Table 2 summarizes RRD features. Clinical and demographic characteristics of control group of healthy eyes are reported in Table 3.

### 3.2. Changes in Iris Perfusion Density

AS-OCTA parameters of the iris were statistically compared at baseline and 1 week after SB. As shown in Table 4, at post-operative 1 week, PD showed a decrease from 66.8 ± 13.2% to 58.55 ± 12.0% in the iris supero-nasal region (*p* = 0.016). On the contrary, no statistically significant differences were observed in all other iris regions (*p* > 0.05).

At 1-month follow-up visit, iris PD significantly decreased in all the analyzed iris regions (Table 5) apart from the superior region (*p* > 0.05) if compared to the baseline. At 6-month follow-up, PD did not show any statistically significant difference in comparison with 1-month values (*p* > 0.05) and was significantly lower in all iris sectors if compared to the baseline except for the superior and superotemporal ones (Table 6).

At one day postoperatively iris image acquisition was excluded from the analysis because of poor quality images due to inability of patients to completely open the eye.

In our cohort of patients no intraoperative and postoperative complications have been detected. No patient needed a second surgery for RRD relapse.

## 4. Discussions

In this study, we analyzed iris vasculature changes occurring in patients with RRD underwent SB surgery, before and after surgical procedure. We found time-related changes in terms of perfusion density. In detail, our results showed a significant reduced iris perfusion for whole 6-month follow-up after SB. Of note, a further topographical iris sub-analysis revealed a uniform reduction of the iris vessel network. The latter finding is in agreement with previous histopathological studies illustrating that surgical maneuvers such as encircling scleral buckle, disinsertion of ocular muscles, and diathermy or cryotherapy to the long posterior ciliary vessels may compromise ocular blood flow [9,19,20,21,22,23,24]. Hayreh and Scott [22] published the first in vivo study assessing AS circulation changes after strabismus surgery in humans. Using fluorescein angiography (FA), the authors found significant iris changes after rectus muscles surgery because of an increased incidence of ischemia. Olver and Lee [20] analyzed iris perfusion defects after vertical rectus muscle surgery proving the iris circulation recovered during the first 2 weeks after surgery. Subsequently, by indocyanine green angiography (ICGA), Chan et al. reported similar results showing the ICGA application in detecting perfusion changes of dark irides after strabismus surgery [21].

This paper reports changes in iris perfusion occurring after SB surgery for RRD. Our findings describe as SB surgical technique seems to be associated with a significant reduction in overall iris perfusion at 1 month after surgery and for all 6 months of follow-up. We might speculate that this reduction in perfusion can be secondary to the mechanical stress on the vessels supplying the iris. Moreover, tension of the ocular rectus muscles and the application of a 360° encirclement might cause a serious hypoperfusion allowing to diagnose ASI in the early phase. Using bidirectional laser Doppler technique, Ogasawara et al. measured the retinal arterial circulation following removal of scleral buckling highlighting an increased arterial perfusion due to primarily to the increment in flow velocity [25]. As reported by Diddie and Ernest, removing of encircling bands in rabbit eyes returned the decreased choroidal blood flow to normal levels [26].

OCTA debut has mainly increased the assess of the retinal vasculature, preceding important papers described various applications of OCTA technology for non-invasive imaging of the AS [2,17,27,28,29,30]. Ang et al. [27,28,29] reported corneal and limbal vasculature changes using AS-OCTA providing interesting insights on pathologic corneo-limbal features such as postherpetic scarring and limbal stem cell deficiency [27,28,29]. Notably, AS-OCTA provided quantitative iris vascular density analysis. Over the last years, various authors have described the AS-OCTA technique in iris pathological conditions such as rubeosis iridis and iris racemose hemangiomas [31,32]. Skalet and colleagues evaluated melanocytic iris tumors suggesting an increased iris vascularity as a hall-mark of malignant transformation [33]. Moreover, AS-OCTA has showed a significant sensitivity in identifying small changes in postoperative iris vessel density [17]. In detail, Velez and colleagues examined the iris vascular filing defects, using AS-OCTA, in patients previously treated with strabismus surgery and showed a perfusion decrease in the iris quadrants adjacent to the tensioned rectus muscles [17].

One of the most notable observation from our study was that the iris PD did not show statistically significant differences between baseline and 1-week follow-up visits, apart from supero-nasal region. We might hypothesize that a post-operative inflammatory reaction might account for the absence of changes at an early follow-up. Instead, supero-nasal area of the iris is very difficult to analyze because of eyelid, thus confounding final results.

Our study thus suggests that the AS-OCTA approach may be extremely useful for a fast and noninvasive screening of patients at risk of ASI development in order to avoid more serious complications. Moreover, in post-operative cases, this technique may assist surgeons in diagnosing ASI before clinically manifest symptoms. Therefore, our findings also support the clinical use of AS-OCTA to evaluate the thin structure of the iris and its early vascular disorders. In particular, the assessment of iris perfusion might allow to identify early vasculature alterations in those patients with predisposing factors such as carotid occlusive disease to avoid ASI and to adopt an appropriate vitreoretinal approach [34]. Future studies employing AS-OCTA in iris vascular disorders may shed further light on this aspect.

This paper has some limitations to consider when reading the results. First is the inability to isolate variables and the heterogeneity of patients included. Secondly, AS-OCTA requires participant cooperation, because motion artifacts can influence the images quality. However, AS-OCTA device is still an evolving technology, and the best technological method has to be achieved. The OCTA device used for this study does not provide AS angiography software. Specific AS angiography modules could improve the scan quality. However the low quality images were directly excluded from the analysis. Undoubtedly a longer follow-up would be needed to assess long-term iris changes while we considered a 6-month follow-up. Another limitation of the study is the lack of comparison with a group undergone to cryo buckle surgery without the implant of an encircling band. This could be useful in clarifying whether the 360° band actually plays a role in the iris perfusion changes. Finally, we are unable to understand if the detected changes are secondary to the structural modification secondary surgical procedure rather than to true changes in perfusion. In addition ocular perfusion pressure was not considered at all. To the best of our knowledge, our study is the first description of AS-OCTA in patients undergoing SB. AS-OCTA may be a promising technique for detection and management of anterior segment disorders secondary to uveitis, diabetic retinopathy, iris tumors or ocular surgical procedure. These findings may be relevant for the introduction of novel therapeutic approaches and further analysis of the anterior segment are necessary in order to validate the clinical application proposed in our study.

## 5. Conclusions

Anterior-segment (AS) optical coherence tomography angiography (OCTA) may be a promising technique for detection and management of anterior segment disorders secondary to uveitis, diabetic retinopathy, iris tumors or ocular surgical procedure.

## Figures and Tables

**Table 1:** The clinical characteristics of subjects included in the analysis.

Variables	Description
Age (years)	50.3 ± 17.6
Gender (male/female, %)	9 (60%)/6 (40%)
Axial length	25.2 ± 1.5

**Table 2:** The RRD characteristics of patient included in the analysis.

Refractive Status (*n*, %)	
High myopia (+)	10 (66)
High myopia (−)	5 (33)
**Lens status (*n*, %)**	
Phakia	8 (53)
Pseudophakia	7 (47)
**Number of breaks (mean ± SD (range))**	1.5 ± 1.3 [1,2,3,4,5]
**Extent of retinal detachment (*n*, %)**	
1 quadrant	5 (33)
2 quadrants	6 (40)
3 quadrants	3 (20)
4 quadrants	1 (7)
**Quadrants segmental buckles**	
Superior	4 (26)
Superior-temporal	5 (33)
Inferior	2 (13)
Inferior-nasal	2 (13)
Temporal	2 (13)
**Complication (*n*, %)**	
Secondary glaucoma (IOP > 21 mmHg)	1 (7)
Hypotony (IOP < 5 mmHg)	1 (7)
**IOP mmHg (average)**	
Pre-operative	14 mmHg
Post-operative	18 mmHg

SD: standard deviation; RRD: rhegmatogenous retinal detachment; IOP: intraocular pressure.

**Table 3:** AS-OCTA iris parameters compared in RRD eyes and 15 healthy eyes.

Iris Perfusion Density Baseline			
	RRD Eyes	Healthy Eyes	*p*-Value
Superior	77.8 ± 11.2	77.2 ± 10.9	<0.0001
Supero-temporal	69.5 ± 15.4	68.5 ± 11.9	<0.0001
Supero-nasal	66.8 ± 13.2	67.3 ± 12.0	<0.0001
Temporal	84.4 ± 11.4	86.2 ± 5.7	<0.0001
Nasal	80.2 ± 7.4	81.5 ± 9.6	<0.0001
Inferior	82.0 ± 9.4	79.4 ± 7.6	<0.0001
Infero-nasal	53.6 ± 11.8	54.3 ± 13.5	<0.0001
Infero-temporal	64.0 ± 17.2	65.2 ± 11.9	<0.0001

Data are presented as mean ± SD (standard deviation). AS-OCTA: anterior segment optical coherence tomography angiography; RRD: rhegmatogenous retinal detachment.

**Table 4:** AS-OCTA iris parameters compared at baseline and 1 week after SB.

Iris perfusion density (PD)			
	Baseline	1 Week	*p*-Value
Superior	77.8 ± 11.2	78.2 ± 10.9	>0.050
Supero-temporal	69.5 ± 15.4	70.5 ± 11.9	>0.050
Supero-nasal	66.8 ± 13.2	58.5 ± 12.0	<0.0001
Temporal	84.4 ± 11.4	86.2 ± 6.7	>0.050
Nasal	80.2 ± 7.4	78.5 ± 9.6	>0.050
Inferior	82.0 ± 9.4	82.9 ± 7.6	>0.050
Infero-nasal	53.6 ± 11.8	55.3 ± 13.5	>0.050
Infero-temporal	64.0 ± 17.2	64.9 ± 11.9	>0.050

Data are presented as mean ± SD (standard deviation). AS-OCTA: anterior segment optical coherence tomography angiography; SB: scleral buckling.

**Table 5:** AS-OCTA iris parameters compared at baseline and 1 month after SB.

Iris Perfusion Density (PD)			
	Baseline	1 Month	*p*-Value
Superior	77.8 ± 11.2	68.6 ± 20.5	>0.050
Supero-temporal	69.5 ± 15.4	62.1 ± 9.0	<0.0001
Supero-nasal	66.8 ± 13.2	45.5 ± 8.5	<0.0001
Temporal	84.4 ± 11.4	71.8 ± 22.1	<0.0001
Nasal	80.2 ± 7.4	64.5 ± 16.4	<0.0001
Inferior	82.0 ± 9.4	72.1 ± 17.9	<0.0001
Infero-nasal	53.6 ± 11.8	46.5 ± 14.1	<0.0001
Infero-temporal	64.0 ± 17.2	56.3 ± 11.7	<0.0001

Data are presented as mean ± SD (standard deviation). AS-OCTA: anterior segment optical coherence tomography angiography; SB: scleral buckling

**Table 6:** AS-OCTA iris parameters compared at baseline and 6 months after SB.

Iris Perfusion Density (PD)			
	Baseline	6 Months	*p*-Value
Superior	77.8 ± 11.2	67.2 ± 19.6	>0.050
Supero-temporal	69.5 ± 15.4	61.1 ± 8.2	>0.050
Supero-nasal	66.8 ± 13.2	46.9 ± 9.5	<0.0001
Temporal	84.4 ± 11.4	69.8 ± 19.5	<0.0001
Nasal	80.2 ± 7.4	66.3 ± 18.4	<0.0001
Inferior	82.0 ± 9.4	72.4 ± 18.1	<0.0001
Infero-nasal	53.6 ± 11.8	46.7 ± 14.4	<0.0001
Infero-temporal	64.0 ± 17.2	55.9 ± 10.9	<0.0001

Data are presented as mean ± SD (standard deviation). AS-OCTA: anterior segment optical coherence tomography angiography; SB: scleral buckling.
